# Attenuated *Streptococcus agalactiae* WC1535 ∆Sia perturbs the gut microbiota of *Oreochromis niloticus*, massively colonizes the intestine, and induces intestinal mucosal immunity after intraperitoneal inoculation

**DOI:** 10.3389/fmicb.2022.1036432

**Published:** 2022-11-11

**Authors:** Jingwen Hao, Shuyi Wang, Jicheng Yang, Qianqian Zhang, Zhenbing Wu, Defeng Zhang, Aihua Li

**Affiliations:** ^1^State Key Laboratory of Freshwater Ecology and Biotechnology, Institute of Hydrobiology, Chinese Academy of Sciences, Wuhan, China; ^2^College of Advanced Agricultural Sciences, University of Chinese Academy of Sciences, Beijing, China; ^3^College of Fisheries and Life, Dalian Ocean University, Dalian, China; ^4^Key Laboratory of Fishery Drug Development, Ministry of Agriculture and Rural Affairs, Pearl River Fisheries Research Institute, Chinese Academy of Fishery Sciences, Guangzhou, China

**Keywords:** gut microbiota, immune response, tilapia, *Streptococcus agalactiae*, vaccine

## Abstract

We previously developed and assessed the effectiveness of the attenuated *Streptococcus agalactiae* (Group B *Streptococcus*, GBS) strain WC1535 ∆Sia (with *neuA-D* gene cluster deletion) vaccine in tilapia (*Oreochromis niloticus*). In this study, we characterized the bacterial communities of the tilapia intestines by 16S rRNA high-throughput sequencing and assessed the serum antibody response, expression of immune-related genes, and histological changes following formalin-killed GBS vaccine (FKV) and the live attenuated vaccine ∆Sia (LAV). Results showed that FKV and LAV induced robust systemic and intestinal mucosal immune responses in tilapia without causing obvious pathological changes in the hindgut, spleen, and head kidney but exerted different effects on intestinal bacterial communities. The richness or diversity of the intestinal bacterial community of FKV tilapia showed no significant changes compared with that of the control fish (*p* > 0.05) at either day 21 post-initial vaccination (21 dpiv) or day 35 (day 14 after the second immunization) (35 dpiv). The community composition of FKV tilapia and controls was significantly similar, although the relative abundance of some genera was significantly altered. Relative to control fish, the gut ecosystem of LAV tilapia was significantly disturbed with a substantial increase in community diversity at 21 dpiv (*p* < 0.05) and a significant decrease at 35 dpiv in fish with high serum antibody response (ΔSia35H) (*p* < 0.05). However, there was no significant difference between ΔSia35H and ΔSia35L (low serum antibody response) fish (*p* > 0.05). Moreover, the community composition of LAV tilapia at 21 dpiv or 35 dpiv was considerably different from that of the controls. Particularly, GBS ∆Sia was found to be abundant in the intestine at 21 and 35 dpiv. This result suggested that the parenteral administration of the LAV (∆Sia) may also have the effect of oral vaccination in addition to the immune effect of injection vaccination. In addition, a significant correlation was found between the expression of immune-related genes and certain bacterial species in the intestinal mucosal flora. Our findings will contribute to a better understanding of the effects of inactivated and attenuated vaccines on gut microbiota and their relationship with the immune response.

## Introduction

Numerous bacteria populate the gut and establish a symbiotic microecosystem with the host (Xue et al., 2020), and the homeostasis of the gut microbiota is crucial for the health of the host (Cho and Blaser, 2012). Intestinal dysbiosis is not only accompanied by the occurrence of diseases (Feng et al., 2015; Li et al., 2016, 2017; She et al., 2017; Wang et al., 2018) but is also closely related to metabolic disorders and even brain dysfunction (Lin and Zhang, 2017). Gut microbes can produce a variety of extracellular enzymes that play an important role in host nutrient digestion and absorption (Askarian et al., 2012, 2013; Das et al., 2014; Tran et al., 2017). Evidence suggests that the microbiome and the immune system are closely linked (Hagan et al., 2019). Gut microbes not only participate in immune system development but also play a key role in immune function regulation (Xue et al., 2020). Germ-free (GF) mice had fewer immune cells, lighter thymus, and smaller lymph nodes than normal mice and exhibit Peyer’s patch dysplasia (Bauer et al., 1963; Van Der, 1986; Kim et al., 2013). However, these abnormalities can be reversed after microbial colonization (Yanagibashi et al., 2013; Lin and Zhang, 2017). *Bacteroides fragilis* promoted the production of CD4^+^ T cells *via* polysaccharide A (Mazmanian and Kasper, 2006). Segmented filamentous bacteria aided Th17 cell accumulation in the intestine’s lamina propria (Sczesnak et al., 2011). Certain *Clostridia* members promoted T_reg_ cell proliferation and differentiation *via* regulatory factors, such as TGF-β (Atarashi et al., 2011, 2013). In conventional mice C57BL/6j, broad-spectrum antibiotic therapy for 8 weeks reduced the number of memory T cells, regulatory T cells, and activated dendritic cells in the small intestine, colon, mesenteric lymph nodes, and spleen. The percentages of CD4^+^, CD8^+^, and B220^+^ cells in the small intestine and CD4^+^ cells in the colon recovered 7 days after fecal microbiota transplantation (Ekmekciu et al., 2017). Sturgeon gut microbiota colonizing GF zebrafish guts up-regulated immune-related gene expression (Teame et al., 2020), and similar results were observed with *Bacillus subtilis* (Tan et al., 2019).

Vaccination is a common disease control strategy, and the research on the link between gut microbiota and vaccination has seen some progress in recent years. Through multiomics analysis, Hagan et al. revealed that human immunity to vaccinations was altered by antibiotic-driven gut microbiota disturbance (Hagan et al., 2019). Fecal microbial communities from individuals who received oral typhoid vaccination showed no discernible perturbations, whereas those of individuals displaying multiphasic *Salmonella typhi*-specific cell-mediated immunity showed increased complexity (Eloe-Fadrosh et al., 2013). The TLR5-mediated sensing of flagellin from the microbiota influenced antibody responses to inactivated influenza and polio vaccines (Oh et al., 2014; Rout et al., 2021). The fecal microbiome of responders to rotavirus vaccination in rural Ghana differed from that of nonresponders and resembled that of Dutch infants (Harris et al., 2017). The mouse gut microbiota was unaffected by vaccination with conserved *Escherichia coli* antigens, including MipA, Skp, and ETEC 2479 (Hays et al., 2016). In mice, the intranasal vaccination of lipoprotein SslE followed by two intramuscular injections enhanced the immune response but caused no differences in gut microbial richness or composition compared with the control treatment (Naili et al., 2019). In broiler chickens, different *Salmonella typhimurium* vaccine strains had varying effects on the cecal microbiota but did not affect its relative abundance (Park et al., 2017). The gut bacterial population of grass carp was altered 21 days after vaccination with an oral *Vibrio mimicus* double-targeted DNA vaccine, and gut macrophage activity and hindgut immune-related gene expression were positively linked to *Bacteroides* (Cao et al., 2021). In grass carp, no notable alterations in the dominant genus of the intestinal tract were observed after exposure to recombinant *Aeromonas hydrophila* vaccine (Aera), but the relative abundance of *Aeromonas* changed (Liu et al., 2015). In tilapia (*Oreochromis niloticus*), the oral attenuated *Streptococcus agalactiae* vaccine YM001 altered bacterial community structure in a transient and reversible manner (Li et al., 2018). Notably, the research on gut microbiota and vaccination has mainly focused on humans and mice and mostly involved fecal samples, and studies on fish are rarely reported. Thus, research on fish models may shed new light on the relationship between the gut microbiota and vaccination and may help vaccine development.

Tilapia is an important player in the aquaculture business with annual growth rates of 10–12% (Behera et al., 2018; Acharya et al., 2019; Renuhadevi et al., 2019). GBS is a causal agent of tilapia streptococcosis, which has caused serious economic losses (Laith et al., 2017; Zhang et al., 2017; Li et al., 2019; Liu et al., 2019). Studies have shown that the formalin-killed vaccine (FKV) of GBS protected tilapia against GBS infection for up to 180 days (Pasnik et al., 2005), and our previous work demonstrated that in tilapia, a live attenuated vaccine (LAV ∆Sia) conferred protection against homologous GBS infection (Hao et al., 2022). Although considerable research has been conducted on vaccines for tilapia against GBS infection, a systematic comparison of the effects of FKV and LAV on the hindgut mucosal flora and immune response of tilapia has received little attention. We thus intend to gain insight into how the two above vaccine types affect the intestinal mucosal microbiota and their link to immune response. To the best of our knowledge, this work is the first comprehensive research evaluating the effects of different vaccine types administered *via* intraperitoneal (IP) injection on hindgut mucosal flora, serum antibody responses, hindgut mucosal immunity, and their associations in tilapia.

## Materials and methods

### Strains

The GBS wild-type strain WC1335 used in this study was isolated from the brains of moribund tilapia from a pond in Wenchang City, Hainan Province (Zhang et al., 2019). FKV was prepared with 0.3% formalin from WC1535. Our previous work showed that LAV ∆Sia, a gene-deleted strain of WC1535 obtained through homologous recombination, confers protection against homologous infection in tilapia (Hao et al., 2022).

### Vaccine preparation

The inactivated vaccine was prepared in reference to a previous method with slight modifications (Hayat et al., 2021). The wild-type GBS strain WC1535 stored at −80°C was recovered on brain heart infusion (BHI) agar medium (Becton, Dickinson and Company, United States) and cultured at 28°C overnight. The resulting GBS colonies were subcultured in BHI broth, and log-phase bacterial cells were harvested through centrifugation at 5,000 rpm for 5 min. Sterile phosphate-buffered saline (PBS, 0.01 M, pH 7.2–7.4) was used to wash and resuspend the cells to the concentration of 1.0 × 10^9^ colony forming units (CFU) per milliliter (mL). The bacterial suspension was diluted with buffered formalin (37%) to the final concentration of 0.3% and incubated at 28°C for 48 h at 30 rpm to kill the cells. Formalin-killed bacteria were harvested *via* centrifugation at 5,000 rpm for 5 min, washed three times with sterile PBS, and kept at 2.0 × 10^9^ CFU mL^−1^ at 4°C. In addition, 100 μL of the mixture was cultured on BHI plates for 48 h to determine if the bacteria were still viable. Similarly, LAV ΔSia (Hao et al., 2022) was prepared by being streaked onto BHI agar, cultured, harvested, and adjusted to the final concentration of 2.0 × 10^9^ CFU mL^−1^.

### Experimental design and sample collection

The experiment was conducted at the Mingde Hatching and Breeding Farm in Yingshan County, Hubei Province, China. Three equal-sized cages (1.7 m in length, 0.8 m wide, and 0.65 m deep each) were placed in the same pond for housing the control, FKV, and LAV (∆Sia) groups. The water temperature was kept at approximately 25°C by managing the entry of hot spring water with a valve. Healthy NEW GIFT tilapia weighing 50 ± 6.8 g raised in the same farm were randomly distributed into three groups with 35 fish each and kept for more than 14 days to acclimatize to the pond environment. The spleen tissues of five randomly selected fish were sampled to check whether pathogen infection was present. The fish were fed extruded compound feed from the Haid Group throughout the experiment, and the daily feeding amount was estimated on the basis of 3% of the experimental fish’s body weight (9:00 and 15:00). The fish in the FKV group received an IP injection of 1.0 × 10^9^ CFU fish^−1^ (0.5 mL) formalin-killed WC1535, whereas the fish in the LAV group received an IP injection of 1.0 × 10^9^ CFU fish^−1^ (0.5 mL) LAV WC1535 ∆Sia. The same amount of sterile PBS was administered to the fish in the control group. At 21 days post-initial vaccination (dpiv), four fish were randomly selected from each group and brought back to the laboratory for sample collection. Samples collected from the control, FKV, and LAV groups were designated as the CTRL21 group (C1–C4), FKV21 group (F1–F4), and ∆Sia21 group (S1–S4), respectively. On the same day, the fish in the FKV or LAV group received a second dose of vaccine in the same manner, whereas the control fish received the same volume of sterile PBS. Samples collected at 35 dpiv (14 days after the second immunization) from four fish in the control and FKV groups were designated as the CTRL35 group (C5–C8) and FKV35 group (F5–F8), respectively, whereas samples collected from eight fish in the LAV group were assigned to the ∆Sia35H (high serum antibody response, approximately 1: 4,096, H1–H4) group and ∆Sia35L (low serum antibody response, approximately 1: 64, L1–L4) group in accordance with the individual’s serum antibody agglutination titers.

Before sampling, fish were anesthetized with MS-222 (Sigma, Darmstadt, Germany) solution and wiped with 75% alcohol. Blood was collected from the tail vein with sterile syringes, placed at room temperature for 4 h, then stored at 4°C overnight. The next day, the upper pale-yellow serum fraction was collected through centrifugation at 1,000 × *g* for 10 min and stored at −80°C for later use. The fish were then dissected with sterile scissors to isolate hindgut tissues. The hindgut wall was incised along one side and rinsed repeatedly 3–5 times with sterile PBS after the intestinal contents were scraped off. Subsequently, the PBS-removed hindgut wall was minced, weighed at 0.07 g per tube, and stored at −80°C for 16S rRNA analysis. Moreover, samples of the hindgut, spleen, and head kidney were collected from three randomly selected specimens in each group and preserved in 4% paraformaldehyde universal tissue fixative (Biosharp, China). For RNA extraction, hindgut samples were additionally collected at 35 dpiv (14 days after the second immunization).

### Histopathological observation

The above-mentioned tissue samples that had been fixed with 4% paraformaldehyde were dehydrated, embedded, cut into 4 μm thick sections, and dried at 60°C. Next, the sections were stained with hematoxylin–eosin (HE) solution as briefly described below. First, paraffin sections were dewaxed through treatment with xylene and gradient ethanol. Then, they were placed in Harris hematoxylin for nuclear staining for 3–8 min, separated with 1% hydrochloric acid ethanol for a few seconds, and treated with 0.6% ammonia. The sections were rinsed with running water after each step. Next, they were stained with eosin solution for 1–3 min. Finally, they were completely dehydrated until transparent, then gently air-dried and sealed with neutral gum. The sections were observed under 400× magnification by using a BX53 multispectral imaging system (PEN-MSI-FX, Olympus, United States).

### Antibody agglutination titers

Serum antibody agglutination titers were determined as previously described with some modifications by using 96-well U-bottom microtiter plates (Yun et al., 2020). The procedure is briefly described as follows: First, 120 μL of sterile PBS and 40 μL of serum were added to the wells of the first column, and 80 μL of sterile PBS was added to the other wells of a 96-well microtiter plate. Second, 80 μL of the well-mixed sample was transferred from the first to the second column. The sample was mixed well, and 80 μL of the diluted sample was transferred to the next column. In the same way, serial 2-fold dilutions of serum samples were prepared, and the 80 μL mixture in the last column was discarded. Finally, 20 μL of the GBS antigen cells was added to each well of the microtiter plate and mixed well. GBS WC1535 antigen was prepared in the same way as FKV and adjusted to the concentration of 1.0 × 10^9^ CFU mL^−1^. Sterile PBS buffer served as the negative control for the serum. The microtiter plate was incubated overnight at 4°C after 2 h of incubation at 25°C. On the following day, the agglutination condition at the bottom of the well was checked after allowing the microtiter plate to sit at room temperature for 30 min. If deposits with blurred edges were present on the U-shaped bottom wall, then the sample was judged as positive, and if deposits with clear edges were present on the bottom of the U-shaped bottom wall, then the sample was considered as negative. The serum agglutination titer was defined as the reciprocal of the highest dilution of serum that could be judged to be positive.

### Expression of immune-related genes

On day 14 after the second immunization, hindgut samples were collected, treated with liquid nitrogen, and stored at −80°C. Total RNA was extracted with a TransZol Up Plus RNA Kit (TransGen, China), and cDNA was synthesized from 1 μg of total RNA by using the TransScript All-in-One First-Strand cDNA Synthesis SuperMix (TransGen, China) in accordance with the manufacturer’s instructions. Each 20 μL reaction mix contained 10 μL of PerfectStart® Green qPCR SuperMix (2×) (TransGen, China), 0.4 μL of forward primer (10 μM), 0.4 μL of reverse primer (10 μM), 2 μL of cDNA template, and 7.2 μL of nuclease-free water. Quantitative real-time polymerase chain reaction (qRT-PCR) was performed with a qTOWER3G (Analytik Jena AG, Germany) system with the following procedures: 94°C for 30 s; 39 cycles of 94°C for 5 s, 60°C for 15 s, and 72°C for 10 s. The primers of *β-actin*, *IgM*, *MHC-Iα*, *MHC-IIβ*, *TCR-β*, *CD4*, *CD8α*, *IL-1β*, *IL-8*, and *TNF-α* are presented in Table 1. Three replicates were performed for each sample. Gene expression levels were normalized on the basis of *β-actin* and calculated by using the 2^−ΔΔCT^ method (Livak and Schmittgen, 2001).

**Table 1:** Primers used for qRT-PCR to detect the expression of immune-related genes in posterior intestinal mucosa at 35 dpiv.

Primer	Nucleotide sequence (5′ to 3′)	Accession number	Size (bp)	Reference
β-actin-F	ACAGGATGCAGAAGGAGATCACAG	XM003443127	155	Wangkaghart et al. (2021)
β-actin-R	GTACTCCTGCTTGCTGATCCACAT			
IgM-F	GGATGACGAGGAAGCAGACT	KJ676389	122	Wangkaghart et al. (2021)
IgM-R	CATCATCCCTTTGCCACTGG			
MHC-Iα-F	TTCTCACCAACAATGACGGG	XM026157132	188	Wangkaghart et al. (2021)
MHC-Iα-R	AGGGATGATCAGGGAGAAGG			
MHC-IIβ-F	GAGGAACAAGCTCGCCATCG	JN967618	106	Wangkaghart et al. (2021)
MHC-IIβ-R	AGTCGTGCTCTGACCTCGAG			
TCRβ-F	GGACCTTCAGAACATGAGTGCAGA	HFKV2162889	113	Wangkaghart et al. (2021)
TCRβ-R	TCTTCACGCGCAGCTTCATCTGTT			
CD4-F	TTCAGTGGCACTTTGCTCCTAA	XM031744220	124	Wangkaghart et al. (2021)
CD4-R	TGGGCGATGATTTCCAACA			
CD8α-F	ATGGACCAAAAATGGCTTCTG	XM031747820	118	Wangkaghart et al. (2021)
CD8α-R	GCTGAAAGATCCAATGAATTC			
IL-1β-F	AAGATGAATTGTGGAGCTGTGTT	FF280564	175	Wangkaghart et al. (2021)
IL-1β-R	AAAAGCATCGACAGTATGTGAAAT			
IL-8-F	GCACTGCCGCTGCATTAAG	NM001279704	135	Wangkaghart et al. (2021)
IL-8-R	GCAGTGGGAGTTGGGAAGAA			
TNF-α-F	AGGGTGATCTGCGGGAATACT	NM001279533	119	Wangkaghart et al. (2021)
TNF-α-R	GCCCAGGTAAATGGCGTTGT			

### Library preparation and Illumina Miseq sequencing

The total DNA of all samples was extracted by using the DNeasy® Blood & Tissue Kit (Qiagen, Germany) in accordance with the manufacturer’s instructions and detected through 1% agarose gel electrophoresis. The barcoded-primers 338F (5′-ACTCCTACGGGAGGCAGCAG-3′) and 806R (5′-GGACTACHVGGGTWTCTAAT-3′) (Liao et al., 2022) were used to amplify the V3–V4 region of the 16S rRNA gene, and PCR amplification was performed on ABI Genemp®9,700 by using TransStart Fastpfu DNA polymerase (TransGen, China). Each 20 μL reaction mix contained 10 μL of pro-Taq (2×), 0.8 μL of forward primer (5 μM), 0.8 μL of reverse primer (5 μM), template DNA, and ddH_2_O. The PCR parameters were 1 × (95°C, 3 min); 29 × (95°C, 30 s; 53°C, 30 s; 72°C, 45 s); 1 × (72°C, 10 min). Each sample was amplified in triplicate. The PCR products were purified by using an AxyPrep DNA Gel Recovery Kit (Axygen, United States), eluted with Tris–HCl, detected through 2% agarose gel electrophoresis, and quantified by using a QuantiFluor™-ST blue fluorescence quantitative system (Promega, Beijing). All amplicons were sequenced on an Illumina Miseq platform by Shanghai Majorbio Bio-pharm Technology Co., Ltd.

### Data processing, bioinformatics, and statistical analyses

The paired-end reads obtained from the Illumina Miseq platform were merged by Flash (version 1.2.11)^1^ and filtered by Trimmomatic on the basis of the following methods and parameters: (1) Read bases with tail quality values below 20 were filtered, and reads were truncated if the average quality values in the 50 bp window were below 20. Short truncated reads (less than 50 bp) and reads containing N-base were not retained. (2) Paired-end reads with an overlapping length of at least 10 bp were merged into one sequence. (3) A mismatch ratio of 0.2 was allowed for the overlapping regions of the merged sequences. (4) Samples were differentiated, and sequence orientation was corrected in accordance with the barcodes and primers at the beginning and end of the sequences. Primers can have up to two mismatches, whereas barcodes cannot have mismatches. The obtained sequences were processed by using USEARCH (version 11)^2^ as follows: Nonrepetitive sequences were extracted from optimized sequences^3^; single sequences without repeats were removed (see text footnote 3); operational taxonomic units (OTUs) were clustered by using UPARSE (version 11, see text footnote 2) at 97% similarity, and representative sequences of OTUs were obtained after chimeras were removed; and all sequences were mapped to the OTU representatives to obtain strain abundances. Representative OTU sequences were classified by using the RDP classifier (version 2.2)^4^ against the bacterial Silva reference database (Release138)^5^, and the confidence threshold was defined as 0.7.

Alpha diversity was evaluated on the basis of the Ace and Shannon indices, and the Wilcoxon rank-sum test was used to determine significant differences at 95% confidence intervals. Beta diversity was estimated on the basis of principal coordinates analysis (PCoA) and hierarchical clustering analysis (average linkage). Differences in community composition at the genus level were analyzed through the Wilcoxon rank-sum test, and the *p*-values were corrected with the false discovery rate method (*p* < 0.05). Linear discriminant analysis (LDA) effect size (LEfSe) (all-against-all) was performed to identify differentially abundant OTUs on the basis of the nonparametric Kruskal–Wallis H test, and LDA was used to determine the effect size of differentially abundant taxa. Phylogenetic Investigation of Communities by Reconstruction of Unobserved States (PICRUSt2) was applied to predict the functional profiles of microbial communities by using 16S rRNA gene data (Douglas et al., 2020). The statistical analysis of taxonomic and functional profiles (STAMP) was performed to process the functional profiles of gut microbial communities predicted by PICRUSt2, and Welch’s *t*-test (Bonferroni multiple test correction) was conducted to identify the metabolic pathways at Kyoto Encyclopedia of Genes and Genomes (KEGG)^6^ level 3 that differed significantly between the sets of profiles of the two groups (Parks et al., 2014). Differences in serum antibody agglutination titers between groups were determined through one-way ANOVA followed by Dunnett’s T3 (3) *post hoc* tests by using SPSS 18.0 (SPSS, Chicago, United States). Differences in immune-related gene expression between groups were determined through one-way ANOVA followed by LSD *post hoc* tests by using SPSS 18.0. Spearman correlation analysis was conducted to evaluate the association of immune-related gene expression with microbial members at the order level.

## Results

### Overview of the study and sequence data

**Figure 1:**
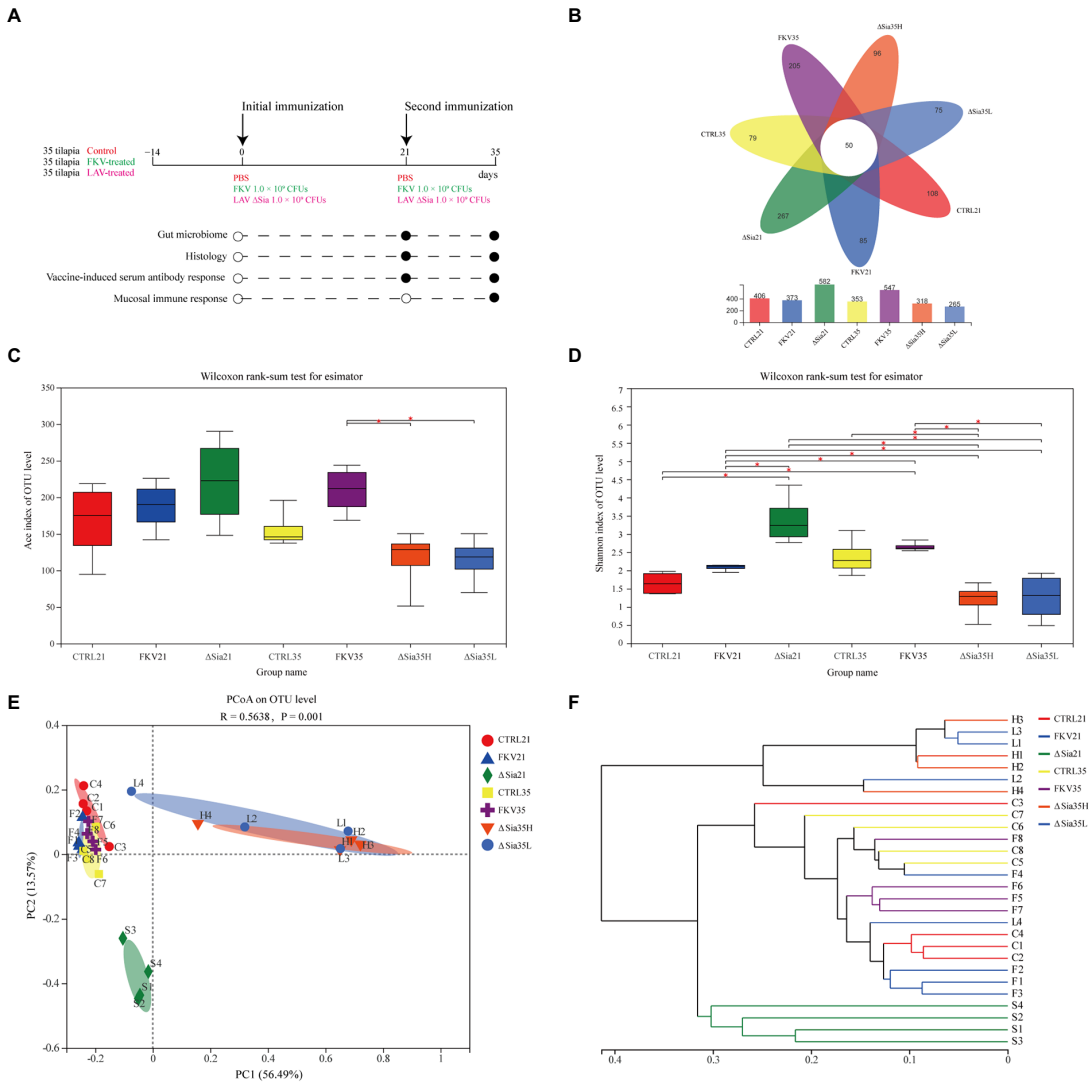
Study overview and the effects of FKV and LAV ∆Sia on the gut bacterial community of healthy tilapia. **(A)** Study overview. A total of 105 healthy NEW GIFT tilapia weighing 50 ± 6.8 g were randomly divided into three groups with 35 fish each and acclimatized for more than 14 days. The fish in the vaccinated group received the first dose of FKV or LAV through intraperitoneal administration on day 0 and the second dose on day 21, while the fish in the control group received the same amount of sterile PBS. Tissue and serum samples were collected on days 21 and 35, and analyses were performed as shown in the plot (black circles). **(B)** Venn diagram depicting shared or unique OTUs in different groups. **(C**,**D)** Alpha diversity index **(C)** Ace reflects community richness, and **(D)** the Shannon index shows community diversity in each group. Box plots display median values and interquartile ranges. An asterisk (*) indicates statistical differences at *p* < 0.05. **(E)** PCoA of bacterial communities based on the OTU level. The PCoA of sample community composition was performed on the basis of the Bray–Curtis algorithm with the permutation number of 999. The scales of the horizontal and vertical axes show relative distances, and percentages represent contributions to differences in community composition. Close distances between two sample points indicate similar community compositions. **(F)** Analysis of hierarchical clustering based on the OTU level. The sample community distance matrix based on the Bray–Curtis algorithm is clustered by using the average clustering method, and the length of the branches represents the distance between samples. CTRL21 (C1–C4), control group at 21 dpiv; FKV21 (F1–F4), FKV group at 21 dpiv; ∆Sia21 (S1–S4), LAV ∆Sia group at 21 dpiv. CTRL35 (C5–C8), control group at 35 dpiv; FKV35 (F5–F8), FKV vaccine group at 35 dpiv (14 days after the second immunization); ∆Sia35H (H1–H4), LAV ∆Sia group with a high serum antibody response at 35 dpiv (14 days after the second immunization); ∆Sia35L (L1–L4), LAV ∆Sia group with a low serum antibody response at 35 dpiv.

A total of 105 healthy NEW GIFT tilapia weighing 50 ± 6.8 g were randomly assigned to three groups of 35 and subjected to more than 14 days of acclimatization. Fish in the vaccine group received the first dose of FKV or LAV through IP injection on day 0 and the second dose on day 21. Fish in the control group received the same volume of sterile PBS. Tissue and serum samples were collected on days 21 and 35, and analyses were performed as shown in Figure 1A.

The number of effective sequences per sample was 34,216–76,146 with an average length of 399–428 bp (Supplementary Table 1). The valid sequences of all samples were normalized to 30,185 reads, and 1,472 OTUs were obtained on the basis of 97% similarity. The representative sequences of all OTUs were classified into 37 phyla, 92 classes, 214 orders, 357 families, 665 genera, and 973 species. As shown in Supplementary Figure 1 and Table 2, the rarefaction curve based on the OTU level tended to approach the asymptote, and the Good’s coverage of all samples exceeded 99%, indicating that the sequence database was sufficient to reflect the information of almost all the microbial diversity in the samples. As shown in the Venn diagram (Figure 1B), 50 OTUs were shared among different groups, and the number of OTUs unique to the CTRL21, FKV21, ∆Sia21, CTRL35, FKV35, ∆Sia35H, and ∆Sia35L groups was 108, 85, 267, 79, 205, 96, and 75, respectively.

**Table 2:** Alpha diversity indices and difference analysis of the gut microbiota in different groups.

Group	Alpha diversity indices					
	Chao	Ace	Sobs	Shannon	Simpson	Coverage
CTRL21	164.27 ± 56.55^a^	165.97 ± 56.47^a^	146.75 ± 50.54^a^	1.65 ± 0.33^a^	0.35 ± 0.04^a^	1.00^a^
FKV21	184.67 ± 34.32^a^	187.14 ± 36.77^a^	155.25 ± 29.35^a^	2.08 ± 0.09^a^	0.10 ± 0.05^b^	1.00^a^
∆Sia21	221.53 ± 65.45^a^	220.90 ± 65.27^a^	212.50 ± 64.96^a^	3.40 ± 0.70^b^	0.10 ± 0.05^b^	1.00^a^
CTRL35	159.90 ± 22.63^a^	156.31 ± 26.74^a^	140.00 ± 27.33^a^	2.38 ± 0.53^a^	0.18 ± 0.05^a^	1.00^a^
FKV35	212.91 ± 31.42^a^	209.11 ± 34.42^a^	199.25 ± 33.50^a^	2.65 ± 0.13^a^	0.18 ± 0.01^a^	1.00^a^
∆Sia35H	118.23 ± 47.03^a^	114.62 ± 43.50^a^	106.50 ± 39.24^a^	1.19 ± 0.48^b^	0.58 ± 0.20^b^	1.00^a^
∆Sia35L	119.40 ± 34.21^a^	114.20 ± 33.59^a^	100.50 ± 35.12^a^	1.26 ± 0.68^a^	0.53 ± 0.28^b^	1.00^a^

The same superscript letter indicates no significant difference, and different superscript letters indicate a significant difference compared with the corresponding control group at the 0.05 level (Wilcoxon rank-sum test).

CTRL21, control group at 21 dpiv; FKV21, FKV group at 21 dpiv; ∆Sia21, LAV ∆Sia group at 21 dpiv. CTRL35, control group at 35 dpiv; FKV35, FKV vaccine group at 35 dpiv (14 days after the second immunization); ∆Sia35H, LAV ∆Sia group with a high serum antibody response at 35 dpiv (14 days after the second immunization); ∆Sia35L, LAV ∆Sia group with a low serum antibody response at 35 dpiv.

### Analysis of the diversity and similarity of intestinal mucosal flora

We compared the Ace richness and Shannon diversity indices of bacterial communities in samples of tilapia hindgut mucosa collected at 21 and 35 dpiv to assess the effect of the two vaccine candidate strains on gut microbial communities. As shown in Figures 1C,D and Table 2, the Ace and Shannon indices of the FKV21 and FKV35 groups did not significantly differ from those of their respective control groups (Wilcoxon rank-sum test; *p* > 0.05). Although the ΔSia21, ΔSia35H, and ΔSia35L groups showed no significant differences in Ace indices compared with the control group at the same time point (Wilcoxon rank-sum test; *p* > 0.05), the Shannon index of the ΔSia21 and ΔSia35 (ΔSia35H and ΔSia35L) groups showed the opposite changes. The Shannon index of the ΔSia21 group was significantly higher than that of the CTRL21 and FKV21 groups (Wilcoxon rank-sum test; *p* < 0.05), whereas that of the ΔSia35H group was significantly lower than that of the CTRL35 group (Wilcoxon rank-sum test; *p* < 0.05). The Shannon index of the ΔSia35L group was also lower than that of the CTRL35 group, but there were no significant differences between them (Wilcoxon rank-sum test; *p* > 0.05). Moreover, the Shannon indices of the ΔSia35H and ΔSia35L groups showed no significant differences (Wilcoxon rank-sum test; *p* > 0.05).

Beta diversity was used to assess the overall similarity of bacterial communities between groups. Analysis of similarity (ANOSIM) found that between-group differences were significantly larger than within-group differences (R = 0.5638, *p* = 0.001) (Supplementary Table 2). The PCoA plot based on Bray–Curtis distance depicted the beta diversity, which accounted for 70.06% of the variations in sample community structure. The samples from the CTRL21, FKV21, CTRL35, and FKV35 groups clustered closely but separately from those from the ∆Sia21, ∆Sia35H, and ∆Sia35L groups (Figure 1E). The hierarchical clustering tree, which was constructed through the unweighted pair-group method with arithmetic means based on the Bray–Curtis distance, illustrated that the samples from the CTRL21, FKV21, CTRL35, and FKV35 groups clustered together then clustered with the ∆Sia21 samples, whereas the samples of the ∆Sia35H and ∆Sia35L groups, except for sample L4, clustered into a single branch (Figure 1F).

### Composition of bacterial communities

**Figure 2:**
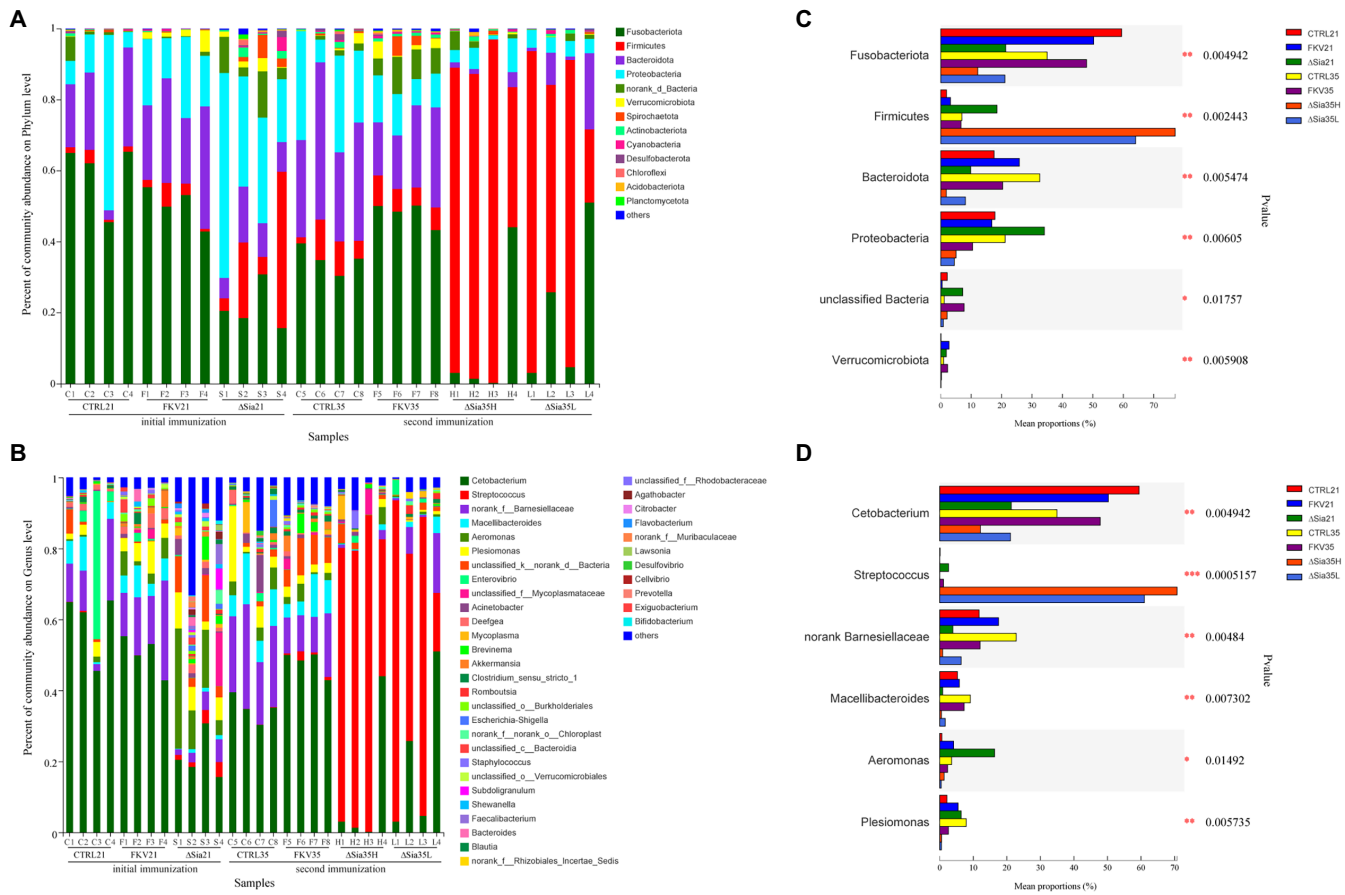
Community composition of samples from various groups at the **(A)** phylum level and **(B)** genus level. The horizontal axis shows the sample names, and the vertical axis shows the relative abundance of species. Species with abundance ratios of less than 0.01 in all samples were classified as others. Mean proportion of dominant phyla **(C)** and genera **(D)** in different groups. CTRL21 (C1–C4), control group at 21 dpiv; FKV21 (F1–F4), FKV group at 21 dpiv; ∆Sia21 (S1–S4), LAV ∆Sia group at 21 dpiv. CTRL35 (C5–C8), control group at 35 dpiv; FKV35 (F5–F8), FKV vaccine group at 35 dpiv (14 days after the second immunization); ∆Sia35H (H1–H4), LAV ∆Sia group with a high serum antibody response at 35 dpiv (14 days after the second immunization); ∆Sia35L (L1–L4), LAV ∆Sia group with a low serum antibody response at 35 dpiv.

We further examined the community composition to understand the effects of vaccination on bacterial communities further. As shown in Figures 2A,C, in all experimental groups, the dominant phyla (relative abundance >1%) in the gut bacterial community of tilapia were Fusobacteriota, Firmicutes, Proteobacteria, Bacteroidetes, norank_d_Bacteria, Verrucomicrobiota, Spirochaetota, Actinobacteriota, Desulfobacterota, Cyanobacteria, Chloroflexi, Acidobacteriota, and Planctomycetota. Although variations in the relative abundances between groups were found, the intestines of the tilapia in the control groups (CTRL21 and CTRL35) and the inactivated vaccine groups (FKV21 and FKV35) had similar dominant phyla, including Fusobacterium, Bacteroidetes, and Proteobacteria. In the gut microbiota of tilapia in the ∆Sia21 group, the four most dominant taxa were Proteobacteria, Fusobacterium, Firmicutes, and Bacteroidetes in that order. Firmicutes, Fusobacterium, Bacteroidetes, and Proteobacteria predominated in the gut of the tilapia in the ∆Sia35H and ∆Sia35L groups.

As exhibited in Figures 2B,D, at the genus level, the CTRL21 group was dominated by *Cetobacterium*, norank Barnesiellaceae, *Macellibacteroides*, and *Enterovibrio*; the FKV21 group was dominated by *Cetobacterium*, norank Barnesiellaceae, *Macellibacteroides*, *Aeromonas*, and *Plesiomonas*; and higher proportions of *Cetobacterium*, *Aeromonas*, *Plesiomonas*, and *Acinetobacter* were observed in the ∆Sia21 group than in other groups. The dominant genera in the CTRL35 and FKV35 groups were similar and included *Cetobacterium*, unclassified Barnesiellaceae, *Macellibacteroides*, and *Plesiomonas*. The relative abundance of *Streptococcus*, followed by that of *Cetobacterium*, was the highest in the ∆Sia35H and ∆Sia35L groups. The relative abundances of *Cetobacterium* in the CTRL21 group (59.44% ± 9.2%) and CTRL35 group (35.01% ± 3.82%) were comparable with those in the FKV21 group (50.16% ± 5.28%) and FKV35 group (47.82% ± 3.33%) and higher than those in the ∆Sia21 group (21.36% ± 6.47%), ∆Sia35H group (12.25% ± 21.47%), and ∆Sia35L group (21.11% ± 22.45%). The relative abundance of *Streptococcus* was low in the CTRL21 group (0.13% ± 0.18%), CTRL35 group (0.05% ± 0.07%), FKV21 group (0.02% ± 0.02%), and FKV35 group (1.15% ± 1.01%); rose in the ∆Sia21 group (2.70% ± 1.60%); and dramatically increased in the ∆Sia35H group (70.73% ± 22.09%) and ∆Sia35L group (60.93% ± 33.89%). PCR detection by using the primers neu-dF: CAAAGTTACGCTAGCTATGTGG and neu-dR: AGCATTCAATAGCAGCACTCC revealed that the GBS with a significantly elevated gut abundance in the ΔSia35H and ΔSia35L groups was predominantly a GBS mutant strain (ΔSia) with the PCR product length of 3,347 bp and not the wild-type strain WC1535 with the PCR product length of 6,226 bp (Hao et al., 2022).

### Differences in bacterial communities

**Figure 3:**
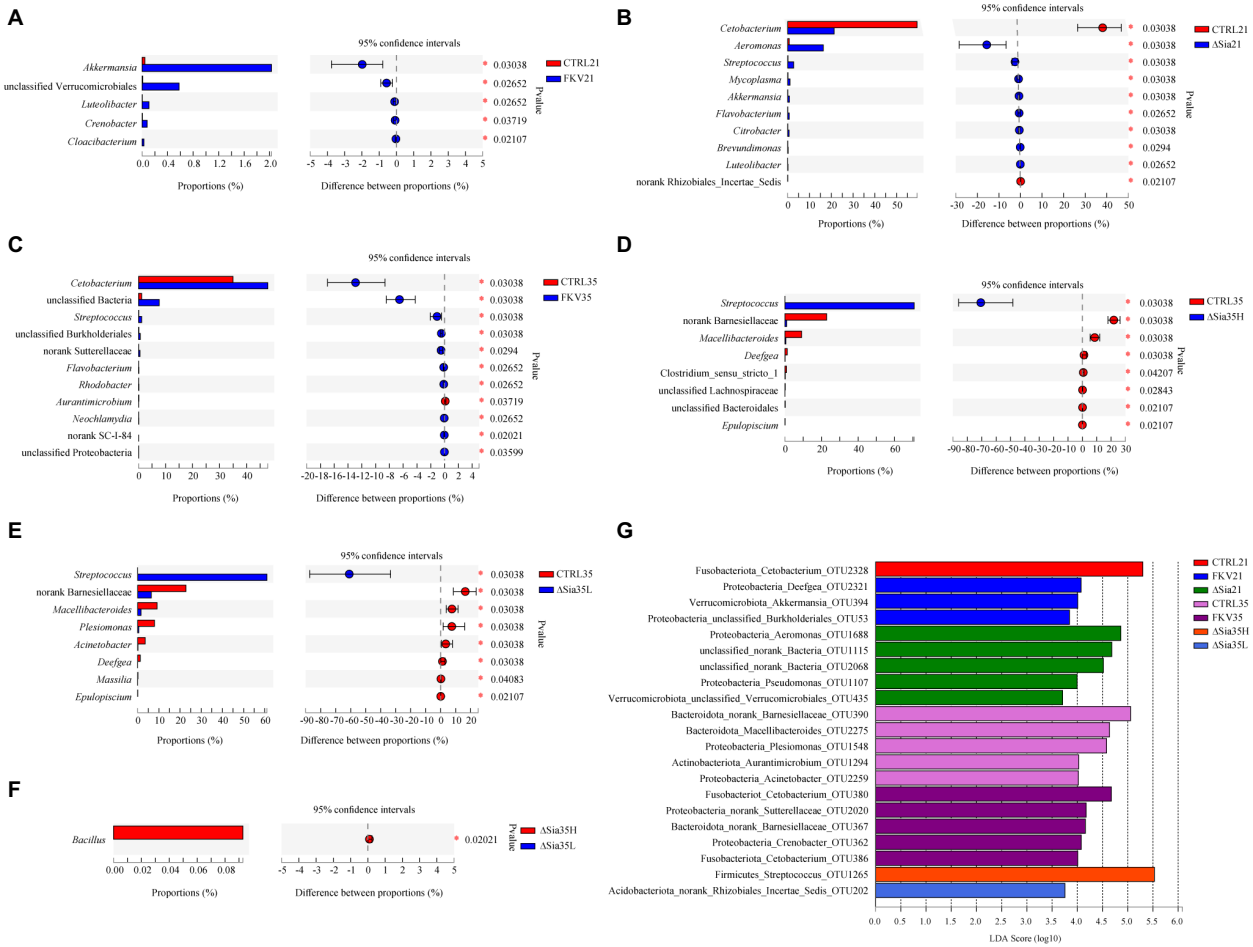
Analysis of differences in community composition at the genus level by the Wilcoxon rank-sum test with 95% confidence intervals (false discovery rate) between **(A)** CTRL21 and FKV21, **(B)** CTRL21 and ∆Sia21, **(C)** CTRL35 and FKV35, **(D)** CTRL35 and ∆Sia35H, **(E)** CTRL35 and ∆Sia35L, and **(F)** ∆Sia35H and ∆Sia35L. **(G)** LEfSe (all-against-all) was performed to identify differentially abundant OTUs among different groups (LDA > 2) on the basis of the nonparametric Kruskal–Wallis H test, and LDA was used to determine the effect size of differentially abundant taxa. The horizontal axis shows the LDA scores, and the vertical axis shows OTUs with significant differences in abundance among different groups. High LDA scores are indicative of the great influence of species abundance on the differential effect. CTRL21, control group at 21 dpiv; FKV21, FKV group at 21 dpiv; ∆Sia21, LAV ∆Sia group at 21 dpiv; CTRL35, control group at 35 dpiv; FKV35, FKV vaccine group at 35 dpiv (14 days after the second immunization); ∆Sia35H, LAV ∆Sia group with a high serum antibody response at 35 dpiv (14 days after the second immunization); ∆Sia35L, LAV ∆Sia group with a low serum antibody response at 35 dpiv.

*Akkermansia*, unclassified Verrucomicrobiales, *Luteolibacter*, *Crenobacter*, and *Cloacibacterium* were enriched in the FKV21 group relative to the CTRL21 group. Compared with the CTRL21 group, the relative abundances of *Cetobacterium* and *Rhizobiales incertae sedis* were significantly decreased in the ∆Sia21 group, whereas those of *Aeromonas*, *Streptococcus*, *Mycoplasma*, *Akkermansia*, *Flavobacterium*, *Citrobacter*, *Brevundimonas*, and *Luteolibacter* were significantly increased. Compared with those in the CTRL35 group, *Cetobacterium*, norank_d_Bacteria, *Streptococcus*, unclassified Burkholderiales, unclassified Sutterellaceae, *Flavobacterium*, *Rhodobacter*, *Neochlamydia*, unclassified SC-I-84, and unclassified Proteobacteria were enriched in the FKV35 group, whereas *Aurantimicrobium* showed the opposite change. The relative abundances of norank Barnesiellaceae, *Macellibacteroides*, *Deefgea*, *Clostridium sensu stricto* 1, unclassified Lachnospiraceae, unclassified Bacteroidales, and *Epulopiscium* in the ∆Sia35H group and norank Barnesiellaceae, *Macellibacteroides*, *Plesiomonas*, *Acinetobacter*, *Deefgea*, *Massilia*, and *Epulopiscium* in the ∆Sia35L group were significantly decreased compared with those in the CTRL35 group, with *Streptococcus* predominating in the ∆Sia35H and ∆Sia35L groups. Moreover, more *Bacillus* was present in the ∆Sia35H group than in ∆Sia35L group (Figures 3A–F).

LEfSe analysis identified 21 differentially abundant OTUs among groups (LDA > 2). *Cetobacterium* OTU2328 was enriched in the CTRL21 group. *Deefgea* OTU2321, *Akkermansia* OTU394, and Burkholderiales sp. OTU53 were enriched in the FKV21 group. *Aeromonas* OTU1688, unclassified sp. OTU1115, unclassified sp. OTU2068, *Pseudomonas* OTU1107, and Verrucomicrobiales sp. OTU435 were enriched in the ∆Sia21 group. Barnesiellaceae sp. OTU390, *Macellibacteroides* OTU2275, *Plesiomonas* OTU1548, *Aurantimicrobium* OTU1294, and *Acinetobacter* OTU2259 were enriched in the CTRL35 group. *Cetobacterium* OTU380, Sutterellaceae sp. OTU2020, Barnesiellaceae sp. OTU367, *Crenobacter* OTU362, and *Cetobacterium* OTU386 were enriched in the FKV35 group. *Streptococcus* OTU1265 was enriched in the ∆Sia35H group, whereas *Rhizobiales incertae sedis* sp. OTU202 was enriched in the ∆Sia35L group (Figure 3G).

### Network analysis

**Figure 4:**
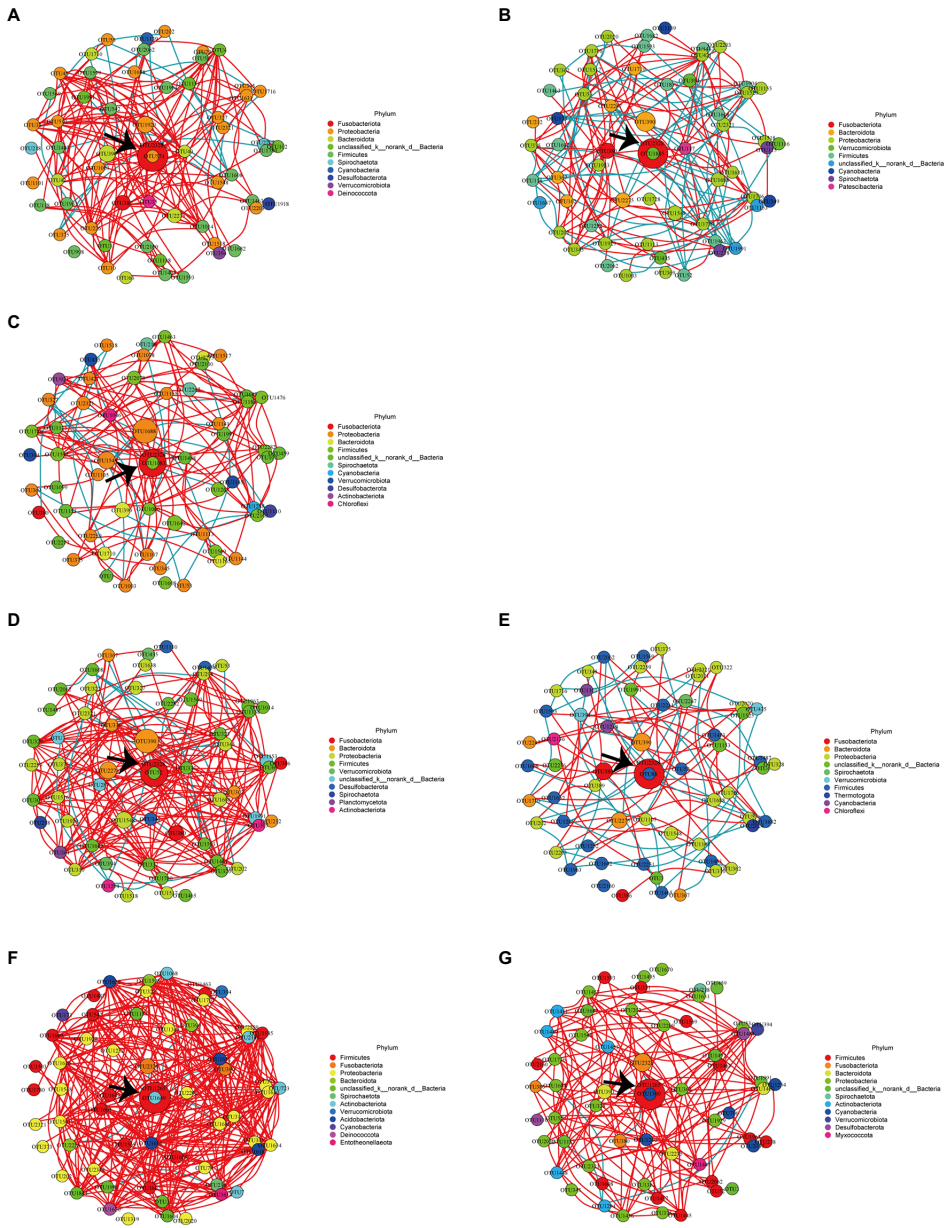
Network analysis of the 60 most abundant OTUs with an absolute Spearman correlation coefficient greater than 0.8 within the **(A)** CTRL21 group, **(B)** FKV21 group, **(C)** ∆Sia21 group, **(D)** CTRL35 group, **(E)** FKV35 group, **(F)** ∆Sia35H group, and **(G)** ∆Sia35L group. Each node represents an OTU; the node’s size reflects abundance, and the node’s color corresponds to a phylum. Red edges indicate positive correlations, whereas blue edges indicate negative correlations. The most abundant OTU in each group is indicated by black arrows. CTRL21, control group at 21 dpiv; FKV21, FKV group at 21 dpiv; ∆Sia21, LAV ∆Sia group at 21 dpiv. CTRL35, control group at 35 dpiv; FKV35, FKV vaccine group at 35 dpiv (14 days after the second immunization); ∆Sia35H, LAV ∆Sia group with a high serum antibody response at 35 dpiv (14 days after the second immunization); ∆Sia35L, LAV ∆Sia group with a low serum antibody response at 35 dpiv.

Network analysis at the OTU level was completed by using Wekemo Bioincloud.^7^ As shown in Table 3, the positive correlation ratios at r > 0 between the OTUs in the FKV groups (FKV21 and FKV35: 50.1 and 50.4%, respectively) and the LAV groups (∆Sia21, ∆Sia35H, and ∆Sia35L: 50.1, 58.0, and 56.7%, respectively) decreased in comparison with that in the control groups (CTRL21 and CTRL35: 57.3 and 59.6%, respectively). Figure 4 shows the interaction between the most abundant 60 OTUs in each group with absolute Spearman correlation coefficients greater than 0.8. Positive correlation ratios in the FKV groups (FKV21 and FKV35: 9.7 and 7.8%, respectively) and the LAV group (11.3% for ∆Sia21) were lower than those in the control groups (CTRL21 and CTRL35: 14.4 and 14.5%, respectively) but increased in the ∆Sia35H group (24.4%) and ∆Sia35L group (16.8%). The core species of the CTRL21, FKV21, CTRL35, FKV35, and ∆Sia21 groups was *Cetobacterium* OTU2328 and that of the ∆Sia35H and ∆Sia35L groups was *S*. *agalactiae* OTU1265.

**Table 3:** Summary of correlations between OTUs within groups based on the Spearman correlation coefficient.

Group	Total edge number	Edge number (*r* > 0)	Ratio ^a^ (*r* > 0)	Total edge number	Edge number (*r* > 0.8)	Ratio ^b^ (*r* > 0.8)
CTRL21	1745	1000	0.573	1740	251	0.144
FKV21	1726	865	0.501	1663	161	0.097
∆Sia21	1749	877	0.501	1680	190	0.113
CTRL35	1727	1030	0.596	1705	432	0.145
FKV35	1729	872	0.504	1708	133	0.078
∆Sia35H	1791	1039	0.580	1755	429	0.244
∆Sia35L	1745	990	0.567	1724	290	0.168

a Represents the ratio of edge numbers with *r* > 0 to the total edge numbers.

b Represents the ratio of edge numbers with *r* > 0.8 to the total edge numbers.

CTRL21, control group at 21 dpiv; FKV21, FKV group at 21 dpiv; ∆Sia21, LAV ∆Sia group at 21 dpiv; CTRL35, control group at 35 dpiv; FKV35, FKV vaccine group at 35 dpiv (14 days after the second immunization); ∆Sia35H, LAV ∆Sia group with a high serum antibody response at 35 dpiv (14 days after the second immunization); ∆Sia35L, LAV ∆Sia group with a low serum antibody response at 35 dpiv.

### Functional prediction of microbial communities and functional profile difference analysis

**Figure 5:**
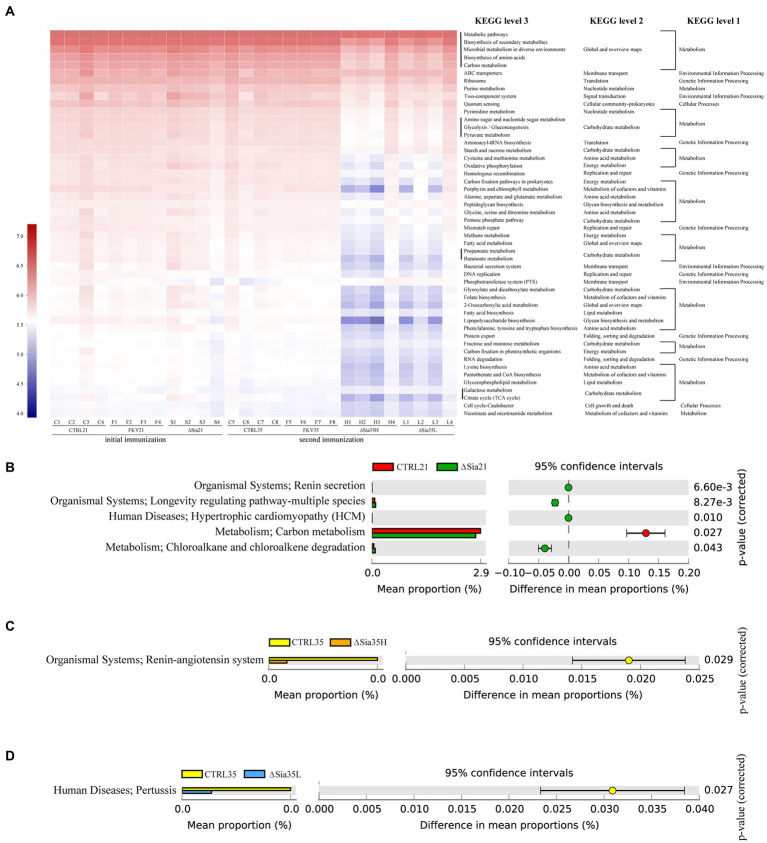
**(A)** KEGG functional prediction of bacterial communities based on PICRUSt2. The horizontal axis represents the sample name, and the vertical axis represents the functional category. Color intensities indicate the abundance of sample functional categories, and legends are shown on the left side of the plot. STAMP was used to process the functional profiles of gut microbial communities predicted by PICRUSt2, and the metabolic pathways at KEGG level 3 that differed significantly between **(B)** CTRL21 and ∆Sia21, **(C)** CTRL35 and ∆Sia35H, and **(D)** CTRL35 and ∆Sia35L were identified through Welch’s *t*-test with a 95% confidence interval (Bonferroni’s multiple test correction). On the basis of same statistical hypothesis testing, no significant differences in functional profiles were found between CTRL21 and FKV21 and between CTRL35 and FKV35. CTRL21 (C1–C4), control group at 21 dpiv; FKV21 (F1–F4), FKV group at 21 dpiv; ∆Sia21 (S1–S4), LAV ∆Sia group at 21 dpiv. CTRL35 (C5–C8), control group at 35 dpiv; FKV35 (F5–F8), FKV vaccine group at 35 dpiv (14 days after the second immunization); ∆Sia35H (H1–H4), LAV ∆Sia group with a high serum antibody response at 35 dpiv (14 days after the second immunization); ∆Sia35L (L1–L4), LAV ∆Sia group with a low serum antibody response at 35 dpiv.

We used PICRUSt2 to map normalized OTU abundances to functional categories at various pathway levels on the basis of KEGG Ortholog information. ANOSIM indicated that the predictive function abundances of the ∆Sia35H and ∆Sia35L groups were separate from those of the CTRL35 and FKV35 groups (Supplementary Table 2). Figure 5A illustrates that metabolic pathways, biosynthesis of secondary metabolites, microbial metabolism in diverse environments, biosynthesis of amino acids, and carbon metabolism were the most abundant functional categories of the bacterial communities in all samples. The abundances of functional categories were considerably lower in the ∆Sia35H and ∆Sia35L groups than in the other groups, including CTRL21, FKV21, CTRL35, and FKV35. These categories included carbohydrate metabolism, amino acid metabolism, replication and repair, polysaccharide synthesis and metabolism, energy metabolism, lipid metabolism, membrane transport, global and overview maps, folding, sorting and degradation, and metabolism of cofactors and vitamins. STAMP revealed no significant differences in the metabolic pathways of the bacterial communities at KEGG level 3 (Welch’s *t*-test, *p* > 0.05) in CTRL21 vs. FKV21, CTRL35 vs. FKV35, and ∆Sia35H vs. ∆Sia35L. Renin secretion, longevity-regulating pathway-multiple species, hypertrophic cardiomyopathy, and chloroalkane and chloroalkene degradation were more abundant and carbon metabolism was less abundant in the ∆Sia21 group than in the CTRL21 group (Welch’s *t*-test, *p* < 0.05). The ∆Sia35H group had a lower abundance of the renin–angiotensin system (RAS) than the CTRL35 group, whereas the ∆Sia35L group had a lower abundance of pertussis (Welch’s *t*-test, *p* < 0.05) (Figures 5C,D).

### Histology, serum antibody response, and mucosal immunity

**Figure 6:**
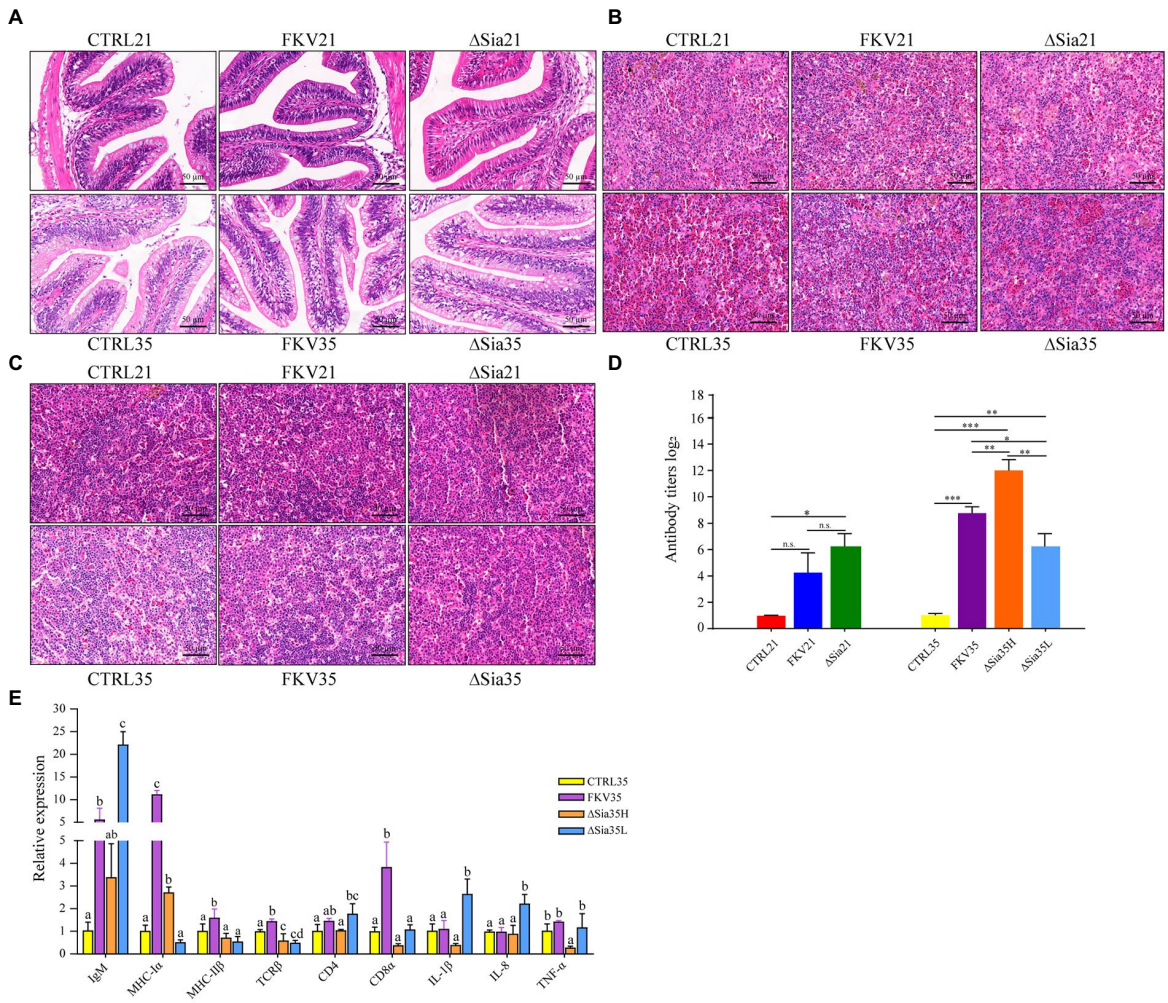
Effects of FKV and LAV vaccination on histology, serum antibody levels, and mucosal immunity-related gene expression in tilapia. **(A)** Representative images of hindgut tissue sections for each group (HE 400×); **(B)** representative images of spleen tissue sections for each group (HE 400×); **(C)** representative images of head kidney tissue sections for each group (HE 400×). **(D)** Serum antibody agglutination titers. Data are presented as mean ± SD (*n* = 4), and the difference between groups was determined through one-way ANOVA followed by Dunnett’s T3 (3) *post hoc* tests by using SPSS 18.0 (SPSS, Chicago, United States). *p < 0.05, **p < 0.01, and ***p < 0.001. n.s., no significance. **(E)** Relative transcriptional levels of immune-related genes in the hindgut mucosa of tilapia at 35 dpiv. The histogram shows the means ± SD of three replicates. Differences in immune-related gene expression between groups were determined by one-way ANOVA followed by LSD *post hoc* tests by using SPSS 18.0. Different letters indicate significant differences between groups at 95% confidence intervals. CTRL21, control group at 21 dpiv; FKV21, FKV group at 21 dpiv; ∆Sia21, LAV ∆Sia group at 21 dpiv. CTRL35, control group at 35 dpiv; FKV35, FKV vaccine group at 35 dpiv (14 days after the second immunization); ∆Sia35H, LAV ∆Sia group with a high serum antibody response at 35 dpiv (14 days after the second immunization); ∆Sia35L, LAV ∆Sia group with a low serum antibody response at 35 dpiv.

Fish in the FKV and LAV groups did not die during the experiment, although they did show decreased appetite. As illustrated in Figure 6, the HE examination of the hindgut, spleen, and head kidney samples revealed no obvious tissue abnormalities in the vaccinated animals. Fish in the FKV group (FKV35) and LAV groups (∆Sia21, ∆Sia35H, and ∆Sia35L) produced significantly higher levels of antibodies than the controls (CTRL21 and CTRL35). The perturbation of the gut ecosystem did not appear to affect LAV fish antibody responses. The effects of WC1535-derived FKV and LAV on immune-related gene expression in the hindgut at 35 dpiv (day 14 after the second immunization) were explored through qPCR. When compared with those in the control, *IgM*, *MHC-Iα*, *MHC-IIβ*, *TCRβ*, and *CD8α* expression levels were significantly increased in the FKV35 group, whereas expression of *CD4*, *IL-1β*, *IL-8*, and *TNF-α* was not significantly changed; the expression of *MHC-Iα* was significantly up-regulated and that of *TCRβ* and *TNF-α* was significantly down-regulated in the ΔSia35H group. The expression levels of *IgM*, *MHC-IIβ*, *CD4*, *CD8α*, *IL-1β*, and *IL-8* were not significantly changed; and the expression levels of *IgM*, *CD4*, *IL-1β*, and *IL-8* were significantly increased and that of *TCRβ* was significantly down-regulated in the ∆Sia35L group.

### Correlation analysis between the top 30 abundant taxa and immune-related gene expression

**Figure 7:**
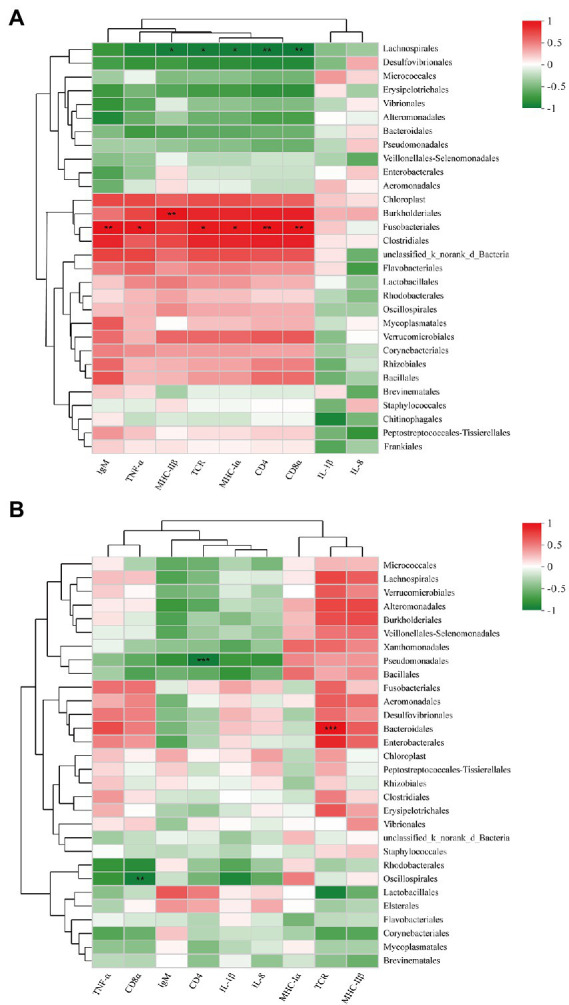
Heatmap showing the correlations of immune-related gene expression with the top 30 abundant taxa at the order level in the **(A)** CTRL35 and FKV35 groups and in the **(B)** CTRL35, ∆Sia35H, and ∆Sia35L groups at 35 dpiv. The horizontal axis represents immune-related gene names, and the vertical axis represents the top 30 abundant taxa. Average-based clustering trees for microbes and genes are shown on the left and top of the plot. The colors in the two-dimensional matrix range from green (negative correlation) to red (positive correlation). The color density represents the magnitude of the correlation coefficient value. The correlation was statistically significant when *r* > 0.8 at *p* < 0.05 (*), *p* < 0.01 (**), or *p* < 0.001 (***).

The relative abundances of species in hindgut bacterial communities and the expression of immune-related genes in the hindgut at 35 dpiv (day 14 after the second immunization) were analyzed on the basis of Spearman correlation coefficients. As shown in Figure 7A, among the top 30 abundant microbes in the CTRL35 and FKV35 groups at the order level, the relative abundance of Lachnospirales was significantly negatively correlated with the expression of *MHC-IIβ* (*r* = −0.833, *p* = 0.010), *TCR* (*r* = −0.833, *p* = 0.0101), *MHC-Іα* (*r* = −0.833, *p* = 0.0101), *CD4* (*r* = −0.857, *p* = 0.0065), and *CD8α* (*r* = −0.857, *p* = 0.0065). A significant positive correlation was found between Fusobacteriales and the expression of *TNF-α* (*r* = 0.826, *p* = 0.0114), *TCR* (*r* = 0.833, *p* = 0.0101), *MHC-Іα* (*r* = 0.833, *p* = 0.0101), *CD4* (*r* = 0.881, *p* = 0.0039), *CD8α* (*r* = 0.881, *p* = 0.0039), and *IgM* (*r* = 0.857, *p* = 0.0065), as well as between Burkholderiales and the expression of *MHC-IIβ* (*r* = 0.857, *p* = 0.0065). As depicted in Figure 7B, among the top 30 abundant microbes in the CTRL35, ∆Sia35H, and ∆Sia35L groups, the relative abundance of Bacteroidales was significantly positively correlated with *TCR* expression (*r* = 0.832, *p =* 0.0008). Pseudomonadales and *CD4* expression (*r* = −0.832, *p =* 0.00079), as well as Oscillospirales and *CD8α* expression (*r* = −0.802, *p =* 0.0019), had significant negative correlations.

## Discussion

Vaccination is a disease control strategy, and vaccine type may affect vaccine efficacy (Gudding and Van Muiswinkel, 2013). A growing body of evidence suggests a close relationship between the gut microbiota and host immunity or vaccine efficiency (Sczesnak et al., 2011; Nakaya and Bruna-Romero, 2015; Harris et al., 2017; Nie et al., 2017; Hagan et al., 2019; Xue et al., 2020). However, a comprehensive investigation of the associations among vaccine types (especially attenuated vaccines), gut microbiota, and vaccine efficiency, especially in tilapia, is lacking. Our results showed that compared with the control fish, tilapia that received one or two doses of FKV had a slightly higher richness and diversity of intestinal bacterial communities at the observed time points and highly similar community compositions. The effect of LAV (∆Sia) on intestinal microbiota was different from that of FKV. The richness and diversity of gut bacterial communities in tilapia increased on day 21 after inoculation with LAV (ΔSia) and decreased on day 14 after booster vaccination. At both time points, the community composition in the LAV tilapia was considerably different from that in the control. General ecological theory holds that high microbial diversity confers ecosystem stability (McCann, 2000; Eloe-Fadrosh et al., 2013), and the administration of FKV and a single dose of LAV (∆Sia) induced community changes in favor of gut microecosystem stability. This change may reflect the beneficial effects of vaccination. Network analysis demonstrated that inoculation with the inactivated or attenuated vaccine (∆Sia) reduced the positive associations between OTUs compared with the control treatment and that highly positive associations (r > 0.8) were strengthened on day 14 after two-dose LAV (∆Sia) administration. We therefore speculated that FKV and single-dose LAV (∆Sia) administration enhanced interspecific competition in the gut microbiota with *Cetobacterium* as the core species, whereas two-dose LAV (∆Sia) administration enhanced high interspecies cooperation with GBS as the core species, especially in the ∆Sia35H group, possibly in response to mucosal colonization by the GBS mutant strain ΔSia. PCR detection and network analysis confirmed that LAV (ΔSia) was able to integrate directly into new communities and alter interspecific interaction patterns in the tilapia gut. Although the number of GBS colonies in the gut increased significantly on day 21 after the tilapia received a single dose of LAV (ΔSia), the relative abundance of GBS (2.70% ± 1.60%) was still within an acceptable range likely due to the resilience of gut microbiota (Li et al., 2018). However, LAV (ΔSia) exhibited considerable enrichment in intestinal mucosa with a relative abundance of up to 70% on day 14 after the booster dose, resulting in a reduction in community diversity. These findings suggest that vaccine type and immune memory may play an important role in shaping the gut microbiota. In addition, Li et al. reported that the gut community diversity in tilapia reached a nadir at 12 h after the oral gavage of GBS strain YM001, with the relative abundance of *Streptococcus* reaching as high as 70.8% and returning to almost its original levels after 15 days (Li et al., 2018). The injection route is commonly accepted to induce a stronger stress response in fish than the oral route. Studies have shown that stress disrupts the body’s homeostasis and affects intestinal physiology; for example, it alters the structure of the intestinal flora, increases gut permeability, and impairs the regenerative capacity of the gastrointestinal mucosa (Bailey et al., 2004, 2010; Konturek et al., 2011). Thus, the stress response induced by the two IP injections may be one of the reasons for the reduction in gut microbial diversity. Overall, the shaping or recovery of gut bacterial communities may be influenced by multiple factors, such as vaccine type, immune memory, stress, vaccination method, genetics, and environment. Additional research is needed to improve our understanding of this phenomenon.

The intestinal bacterial communities were further characterized. The gut microbiota of control tilapia was dominated by the phyla Fusobacterium, Bacteroidetes, and Proteobacteria, and the genera *Cetobacterium*, unclassified Barnesiellaceae, *Macellibacteroides*, and *Enterovibrio*. This finding is different from the results reported by Wu et al., who showed the gut microbiota of tilapia was dominated by the phyla Proteobacteria and Firmicutes and the genera *Undibacterium*, *Escherichia–Shigella*, *Paeniclostridium*, and *Cetobacterium* (Wu et al., 2021). This incongruence may be related to the developmental stage, environment, and diet of the fish (Dusheck, 2002; Galindo-Villegas et al., 2012). Although the overall diversity of the bacterial community in the gut of FKV fish did not change significantly compared with that of the controls, the relative abundance of some species was significantly altered. In particular, the relative abundance of beneficial *Cetobacterium* significantly increased on day 14 following two-dose FKV vaccination. *Cetobacterium* belongs to the phylum Fusobacterium, and its common member *Cetobacterium somerae* is a Gram-negative anaerobic bacterium that colonizes the gastrointestinal tracts of freshwater fish, such as tilapia, common carp, goldfish, and ayu (Finegold et al., 2003; Tsuchiya et al., 2008). *C*. *somerae* efficiently produces vitamin B12, and its abundance is correlated with the demand of fish for this vitamin (Tsuchiya et al., 2008). Moreover, in a zebrafish model, the acetate produced by this bacterium helps improve glucose homeostasis through parasympathetic activation (Wang et al., 2021). The administration of FKV not only resulted in a slight increase in community richness and diversity but also promoted the intestinal colonization of beneficial bacteria, which may be one of the immune-protective mechanisms of conventional vaccines. The abundance of GBS in the intestinal mucosa after the tilapia received the LAV booster reached as high as 70%. This GBS strain was confirmed by PCR to be a mutant GBS (WC1535 ΔSia) strain rather than a wild-type GBS strain, suggesting that the LAV (ΔSia) strain entered and colonized the intestine by some means, thus altering the structure of the original microbiota in the gut. These findings imply that the IP injection of LAV ΔSia may also have the effect of oral vaccination. This situation may be one of the reasons for the typically strong immune response induced by the injectable delivery of attenuated vaccine. The disruption of the gut microbiota is linked to metabolic disturbances (Lin and Zhang, 2017). The functional profiles of the gut microbiota of control and LAV fish, but not those of control and FKV fish, showed substantial variations. Interestingly, presumed renin secretion was high in the ∆Sia21 group, whereas the abundance of RAS in the ∆Sia35H group was reduced compared with that in the control group. The result supports the notion that the gut microbiota modulates physiological and pathological states and that the RAS may be a mediator of gut microbiota effects (Jaworska et al., 2021).

No infectious death occurred during the entire experimental period, and no obvious pathological features were found in the hindgut, spleen, or head kidney of the experimental fish given FKV or LAV (∆Sia), confirming the safety of the two vaccine types. Specific antibody responses and immune-related gene expression are used to assess vaccine efficacy (Adams, 2019; Munang'andu and Evensen, 2019; Kordon et al., 2021). The significant increase in GBS-specific serum antibody levels in the FKV and LAV groups indicated the initiation of humoral immunity. Innate immune-related genes (*IL-1β*, *IL-8*, and *TNF-α*) and adaptive immunity-related genes (*IgM*, *MHC-Iα*, *MHC-IIβ*, *CD4*, *CD8α*, and *TCR*) were examined on day 14 following a booster injection to explore the effect of injectable vaccination with FKV or LAV on intestinal mucosal immunity (Wangkaghart et al., 2021). IgM, the first antibody produced by the immune system, is essential for humoral immunity (Wu et al., 2019). FKV increased the expression of the immune-related genes *IgM*, *MHC-Iα*, *MHC-IIβ*, *TCR-β*, and *CD8α*, indicating that it induced local adaptive immunity. All LAV fish expressed more IgM than the control fish, and fish in the ∆Sia35H group produced less IgM, CD4, CD8α, IL-8, and TNF-α than those in the ∆Sia35L group, implying that the early induction of expression may have been missed. Overall, FKV and LAV (∆Sia) induced systemic and intestinal immunity at the late stage of infection.

The direct effect of microbiota members on immunity is now well understood. *B*. *fragilis* promotes the production of CD4^+^ T cells *via* polysaccharide A, which was beneficial to the host’s immune responses (Mazmanian and Kasper, 2006). Segmented filamentous bacteria reportedly promoted Th17 cell differentiation in a mouse model (Sczesnak et al., 2011). Through regulatory factors, such as TGF-β, certain *Clostridia* members promoted the proliferation and differentiation of T_reg_ cells (Atarashi et al., 2011, 2013). A study on an oral typhoid vaccine targeting human gut flora revealed that the order Clostridiales was associated with cellular immunity in volunteers (Eloe-Fadrosh et al., 2013). The anti-*Shigella* LPS antibody response of cynomolgus macaques had positive correlations with *Weissella*, *Catabacteriaceae*, and *Clostridium* (Seekatz et al., 2013). The expression of immune-related genes in the hindgut of grass carp was positively correlated with *Bacteroides* abundance (Cao et al., 2021). A comparison of RVV responders and non-responders among Ghanaian infants revealed that RVV responses were associated with a decreased abundance of the phylum Bacteroidetes and an increased abundance of *Streptococcus bovis* (Harris et al., 2017). Similarly, on the basis of Spearman correlation analysis, our study showed that intestinal immune-related gene expression was associated with Lachnospirales, Fusobacteriales, Pseudomonadales, Oscillospirales, and Bacteroidales (r-value >0.8, *p* < 0.05). Moreover, individual variability in serum antibody levels were found in experimental fish that received two-dose LAV vaccination, and *Bacillus* was more abundant in the gut microbiota of high LAV responders than in that of other fish. Although the underlying mechanisms through which gut microbes influence vaccine responses remain unclear, their close correlation with mucosal immunity cannot be denied.

In conclusion, FKV and LAV (∆Sia) induced systemic and intestinal mucosal immunity but had different effects on the gut microbiota of tilapia. Inoculation with one or two doses of FKV did not appreciably alter the diversity and composition of bacterial communities. Interestingly, the vaccine strain ∆Sia appeared in large numbers in the tilapia gut after inoculation with an attenuated vaccine and not only altered the structure of the intestinal flora but may also exert the effect of oral vaccination in addition to the immune effect of injection vaccination. Our findings not only provide insight into the relationship between gut microbiota and vaccination but also offer a new perspective for live vaccine development and application.

## Data availability statement

The data presented in the study are deposited in the NCBI repository (http://www.ncbi.nlm.nih.gov/bioproject/876374), accession number PRJNA876374.

## Ethics statement

The animal study was reviewed and approved by the Animal Research Ethics Committee of the Institute of Hydrobiology, Chinese Academy of Sciences.

## Author contributions

JH, SW, JY, QZ, and DZ performed the material preparation and sample collection. JH and ZW performed the data analysis. JH wrote the original draft and revised the manuscript. AL and DZ conceived of the study and revised the manuscript. All authors contributed to the article and approved the submitted version.

## Funding

This study was financially supported by the National Natural Science Foundation of China (No. 32073023), National Key Research and Development Program of China (No. 2020YFD0900300), Key Project of Scientific and Technological Innovation of Hubei Province (2018ABA101), and Guangdong Basic and Applied Basic Research Foundation (2021A1515010956).

## Conflict of interest

The authors declare that the research was conducted in the absence of any commercial or financial relationships that could be construed as a potential conflict of interest.

## Publisher’s note

All claims expressed in this article are solely those of the authors and do not necessarily represent those of their affiliated organizations, or those of the publisher, the editors and the reviewers. Any product that may be evaluated in this article, or claim that may be made by its manufacturer, is not guaranteed or endorsed by the publisher.

## Supplementary material

The Supplementary material for this article can be found online at: https://www.frontiersin.org/articles/10.3389/fmicb.2022.1036432/full#supplementary-material

## Footnotes

^1^ https://ccb.jhu.edu/software/FLASH/index.shtml

^2^ http://drive5.com/uparse/

^3^ http://drive5.com/usearch/manual/singletons.html

^4^ http://sourceforge.net/projects/rdp-classifier/

^5^ http://www.arb-silva.de

^6^ http://www.genome.jp/kegg/

^7^ https://www.bioincloud.tech
