# Supplementary material for: Attenuated Streptococcus agalactiae WC1535 ∆Sia perturbs the gut microbiota of Oreochromis niloticus, massively colonizes the intestine, and induces intestinal mucosal immunity after intraperitoneal inoculation
Source: Front Microbiol. 2022 Nov 11;13:1036432. doi: 10.3389/fmicb.2022.1036432 (PMC9691972; doi:10.3389/fmicb.2022.1036432)
Supplement: Supplementary file 1 [file Data_Sheet_1.docx]

**Supplementary Figure 1.**


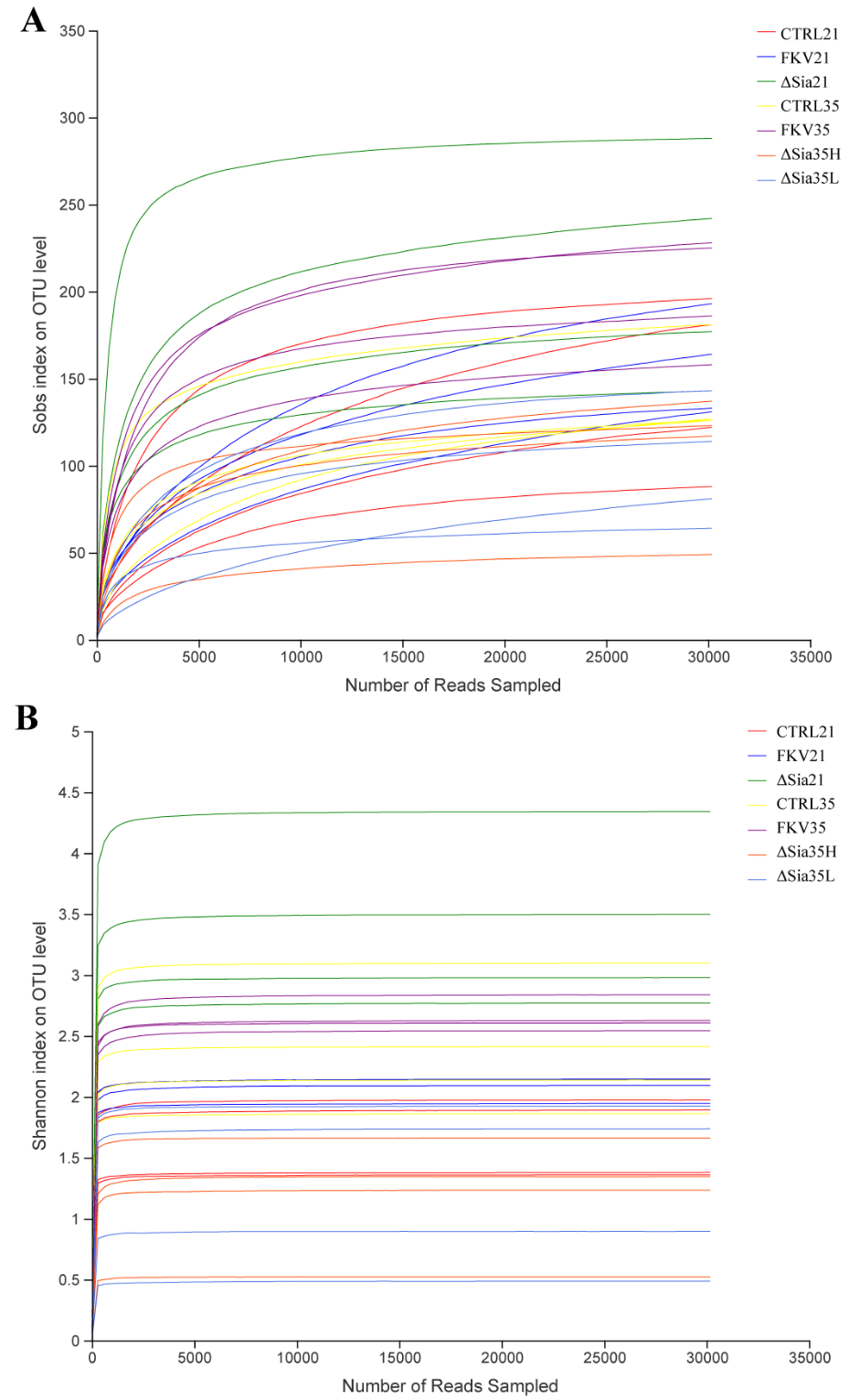


**Supplementary Figure 1.**

Rarefaction curve at the OTU level based on 97% similarity. (**A**) Number of OTUs observed for each sample. (**B**) Shannon curves for each sample. CTRL21: Control group at 21 dpiv; FKV21: FKV group at 21 dpiv; ∆Sia21: LAV ∆Sia group at 21 dpiv. CTRL35: Control group at 35 dpiv; FKV35: FKV vaccine group at 35 dpiv (14 days after the second immunization); ∆Sia35H: LAV ∆Sia group with a high serum antibody response at 35 dpiv (14 days after the second immunization); ∆Sia35L: LAV ∆Sia group with a low serum antibody response at 35 dpiv.

**Supplementary Table 1.** Summary of the sequencing data.

| Sample | Valid reads | Total bases | Mean_length | Min_length | Max_length |
| --- | --- | --- | --- | --- | --- |
| C1 | 34,216 | 13,752,700 | 402 | 200 | 512 |
| C2 | 71,064 | 29,255,786 | 412 | 212 | 507 |
| C3 | 72,298 | 30,183,943 | 417 | 212 | 487 |
| C4 | 63,437 | 26,178,038 | 413 | 213 | 489 |
| F1 | 71,681 | 29,726,300 | 415 | 212 | 493 |
| F2 | 63,775 | 26,431,006 | 414 | 212 | 487 |
| F3 | 76,146 | 31,665,153 | 416 | 233 | 508 |
| F4 | 74,370 | 31,031,086 | 417 | 213 | 499 |
| S1 | 47,286 | 19,515,907 | 413 | 204 | 525 |
| S2 | 58,116 | 23,633,165 | 407 | 206 | 487 |
| S3 | 34,370 | 14,058,615 | 409 | 211 | 532 |
| S4 | 58,339 | 24,112,905 | 413 | 200 | 496 |
| C5 | 74,724 | 31,274,249 | 419 | 212 | 489 |
| C6 | 48,740 | 20,332,175 | 417 | 206 | 497 |
| C7 | 35,821 | 14,794,178 | 413 | 223 | 489 |
| C8 | 52,901 | 21,992,036 | 416 | 204 | 493 |
| F5 | 58,143 | 23,665,788 | 407 | 211 | 516 |
| F6 | 42,597 | 17,005,771 | 399 | 203 | 512 |
| F7 | 40,337 | 16,119,941 | 400 | 202 | 521 |
| F8 | 35,167 | 14,303,565 | 407 | 212 | 502 |
| H1 | 43,712 | 18,288,828 | 418 | 205 | 523 |
| H2 | 59,968 | 25,126,154 | 419 | 212 | 487 |
| H3 | 51,612 | 22,113,421 | 428 | 206 | 431 |
| H4 | 62,992 | 26,274,052 | 417 | 212 | 508 |
| L1 | 69,056 | 29,531,643 | 428 | 223 | 487 |
| L2 | 63,199 | 26,584,517 | 421 | 213 | 432 |
| L3 | 48,140 | 20,368,387 | 423 | 211 | 489 |
| L4 | 62,543 | 25,887,776 | 414 | 211 | 493 |

Note: CTRL21 (C1–C4): Control group at 21 dpiv; FKV21 (F1–F4): FKV group at 21 dpiv; ∆Sia21 (S1–S4): LAV ∆Sia group at 21 dpiv. CTRL35 (C5–C8): Control group at 35 dpiv; FKV35 (F5–F8): FKV vaccine group at 35 dpiv (14 days after the second immunization); ∆Sia35H (H1–H4): LAV ∆Sia group with a high serum antibody response at 35 dpiv (14 days after the second immunization); ∆Sia35L (L1–L4): LAV ∆Sia group with a low serum antibody response at 35 dpiv.

**Supplementary Table 2.** Similarity analysis of the structure and predictive function of gut microbiota in different groups.

| Group | Community structure | | Community function | |
| --- | --- | --- | --- | --- |
|  | R | *p* | R | *p* |
| Whole comparison | 0.564 | 0.001 | 0.366 | 0.001 |
| CTRL21 vs. FKV21 | 0.302 | 0.071 | 0.156 | 0.207 |
| CTRL21 vs. ∆Sia21 | 0.885 | 0.023 | -0.010 | 0.479 |
| FKV21 vs. ∆Sia21 | 0.729 | 0.038 | 0.271 | 0.053 |
| CTRL35 vs. FKV35 | 0.708 | 0.028 | 0.188 | 0.117 |
| CTRL35 vs. ∆Sia35H | 0.906 | 0.031 | 0.708 | 0.022 |
| CTRL35 vs. ∆Sia35L | 0.698 | 0.029 | 0.552 | 0.029 |
| FKV35 vs. ∆Sia35H | 0.875 | 0.026 | 0.719 | 0.024 |
| FKV35 vs. ∆Sia35L | 0.708 | 0.026 | 0.698 | 0.030 |
| ∆Sia35H vs. ∆Sia35L | -0.135 | 0.979 | -0.167 | 0.816 |

Note: ANOSIM analysis compared intra- and intergroup similarities on the basis of the Bray–Curtis algorithm. The R-value ranged from −1 to 1. When R tended to 1, the between-group difference was larger than the within-group difference. The R-value of 0 indicated no difference between groups. When R tended to −1, the between-group difference was smaller than the within-group difference. A permutation test was performed to explore whether the difference was significant. The number of permutations was N = 999, and a *p*-value less than 0.05 indicated a significant difference. CTRL21: Control group at 21 dpiv; FKV21: FKV group at 21 dpiv; ∆Sia21: LAV ∆Sia group at 21 dpiv. CTRL35: Control group at 35 dpiv; FKV35: FKV vaccine group at 35 dpiv (14 days after the second immunization); ∆Sia35H: LAV ∆Sia group with a high serum antibody response at 35 dpiv (14 days after the second immunization); ∆Sia35L: LAV ∆Sia group with a low serum antibody response at 35 dpiv.
